# Exploring the knowledge, attitudes, and perceptions of young men towards fertility in the UK: A cross-sectional survey

**DOI:** 10.1371/journal.pone.0353073

**Published:** 2026-07-10

**Authors:** Jack Tighe, Emeka Okorocha, Anita Mitra, Nicholas Anson, Benjamin P. Jones, Rachel Roberts, Muna Elshieikh, Efstathios Theodorou, Jara Ben Nagi, Lorraine S. Kasaven

**Affiliations:** 1 Department of Gynaecology, Hammersmith Hospital, Imperial College Healthcare NHS Trust, London, United Kingdom; 2 Fertility Unit, Centre for Reproductive and Genetic Health, London, United Kingdom; 3 Department of Emergency Medicine, East Kent Hospitals NHS Trust, Kent, United Kingdom; 4 Department of Gynaecology Oncology, Watford General Hospital, West Hertfordshire Teaching Hospitals NHS Trust, Hertfordshire, Watford, United Kingdom; 5 Department of Surgery and Cancer, Institute of Reproductive & Developmental Biology, Imperial College London, Hammersmith Hospital Campus, London, United Kingdom; 6 The Lister Fertility Clinic, The Lister Hospital, London, United Kingdom; 7 Department of Metabolism, Digestion and Reproduction, Institute of Reproductive & Developmental Biology, Imperial College London, Hammersmith Hospital Campus, London, United Kingdom; 8 Department of Obstetrics and Gynaecology, Danat Al Emarat Hospital, Abu Dhabi, United Arab Emirates; 9 Assisted Conception Unit, Guy’s Hospital, Guy’s and St Thomas’ NHS Foundation Trust, London, United Kingdom; University Hospital of Münster, GERMANY

## Abstract

**Introduction:**

Across many countries, paternal age at childbirth is rising, increasing exposure to age-related fertility decline. This study evaluated fertility-related knowledge, attitudes, and perceptions among young men.

**Methods:**

An anonymous cross-sectional survey was distributed via social media between 13/02/2025 and 29/09/2025. UK adult males completed 54 close-ended questions covering participant characteristics, fertility knowledge, reproductive aspirations, and personal/socioeconomic influences.

**Results:**

A total of 408 men participated in the survey, of whom 214 (52.5%) completed all components, with a median age of 34 years (interquartile range 31–38). Most were married (61.5%; N = 176), had degree-level education or higher (78.4%; N = 226), and were in full-time employment (85.4%; N = 246). Over half had no children (55.6%; N = 160). Most wished to have their first child between 30–35 years (64.5%; N = 71), and last between 36–40 years (64.3%; N = 119).

More than half of the participants recognised that paternal age affects fertility (58.6%; N = 133). However, few were aware of the contribution of male-factor infertility (16.9%; N = 38), or the age at which semen quality declines (17.7%; N = 40). The majority understood that modifiable lifestyle factors influence semen quality (88.9%; N = 200), and either agreed or strongly agreed that smoking (99.1%; N = 211), recreational drug use (95.3%; N = 203), and radiation/chemical exposure (98.1%; N = 209) impacted male fertility.

Many men (43.2%; N = 92) had never sought fertility information; among those who did, online medical websites were most common (32.9%; N = 70), while social media was rarely used (1.4%; N = 3). Key considerations influencing reproductive plans included biological parenthood (76.5%; N = 160), partner suitability (94.7%; N = 196), financial security (95.2%; N = 197), and suitable housing (72.4%; N = 150).

**Conclusion:**

Young men demonstrated limited awareness of age-related fertility decline despite recognising lifestyle effects. Routine healthcare offers an opportunity for counselling to improve awareness, alongside consideration of socioeconomic factors. Findings are limited by a small, highly educated sample and possible self-selection bias.

**Trial registration number:**

Imperial College Research Ethics Committee number: 700608.

## Introduction

Infertility is estimated to impact 17.5% of couples worldwide [1,2], with male factor causes accounting for approximately 30% of all cases [3,4]. Since the 1970s, global fertility rates have shown a steady decline, falling from 4.08 children per woman in 1975 to 2.32 in 2021, although a degree of geographic variation remains [5]. Concurrently, the age of first-time motherhood has risen to 30.9 years-old in 2023 in the UK, compared to 26.7 years-old in 1970, with similar trends reported across Europe [6–8]. Such trends have previously been attributed to advancements in gender equality altering female reproductive aspirations [9]. Women have reported choosing to delay starting a family for a multitude of reasons, including economic security, educational opportunities, and career progression, although the most commonly reported reason is the absence of a partner [10]. However, despite similar trends being observed amongst men, there is a paucity of literature exploring the knowledge, attitudes, and perceptions of fertility reflective of the views of men living in the twenty-first century [11,12].

Male age has been associated with changes in reproductive potential, including alterations in semen quality, and an increased risk of genetic disorders in offspring of older men [13,14]. While advancing paternal age has also been linked to reduced fertilisation and live birth rates, interpretation of these associations is complicated by the strong correlation between male and female partner age, with female age recognised as a major determinant of reproductive outcomes [15,16]. In the UK, mean paternal age at childbirth has gradually increased, with an average age of 33.7 years-old in 2022 compared to 29.4 years-old in 1974 [17]. Consequently, societal changes in paternal age at childbirth may lead to an increased risk of involuntary childlessness, and an increased utilisation of fertility treatments. Previous surveys have highlighted that men have significant gaps in their understanding of female fertility, underestimating the impact of age-related fertility decline (ARFD) on women, and overestimate the success of assisted reproductive technologies (ART) [18–20]. However, few studies have explored men’s knowledge and attitudes towards their own fertility, representing a potentially important, yet often missed opportunity to improve patient education and counselling amongst this population.

Although several studies have examined fertility awareness and attitudes in the general population, participants have been predominantly female and have typically responded to knowledge-based items focused specifically on female fertility [21–23]. In studies that included both men and women, male participants represented only a small proportion of the sample, and were often drawn from university populations, limiting the generalisability of the findings [20]. Population-based studies assessing men’s fertility knowledge have also been subject to selection bias, as surveys were restricted to childless men and those currently in a relationship [24,25].

The most recent survey of young men’s fertility knowledge, conducted in 2015, included 701 male participants and examined their understanding of factors influencing reproductive potential [26]. Although this study explored male fertility knowledge in greater depth than earlier work, several limitations remain [26]. Firstly, while it assessed awareness of determinants of fertility, it did not sufficiently address knowledge of male reproductive physiology, which represents an important component of patient education and counselling. Furthermore, although participants were asked about their desire for future children, the study did not provide an in-depth exploration of men’s reproductive aspirations, limiting its relevance to broader public health and family-planning initiatives. Moreover, this study did not investigate the personal or socioeconomic factors shaping reproductive aspirations, which could help elucidate the underlying drivers of shifts in paternal age and family-planning behaviours [11,12,26]. Finally, as this survey is now approximately a decade old, its conclusions may no longer reflect contemporary knowledge or attitudes towards male fertility. Collectively, these limitations highlight the need for current evidence examining male fertility knowledge and reproductive aspirations in greater depth, to better inform counselling and health-education strategies.

The aim of this study was to provide an up-to-date and in-depth examination of young men’s knowledge, attitudes, and perceptions regarding fertility in the UK. To address this objective, we developed and distributed an anonymous online survey comprising 54 close-ended questions. We hypothesised that young men may underestimate the role of male fertility in determining overall reproductive potential, particularly the extent to which fertility declines with increasing paternal age, as well as the influence of other lifestyle, environmental, and health-related factors known to affect male fertility.

## Materials and methods

An anonymous online survey on young men’s knowledge, attitudes, and perceptions of fertility in the UK was designed using the Qualtrics® platform. The initial survey was designed based on previous reproductive medicine surveys, population-based studies, and national census data [5,10,17,23,27,28]. The draft questionnaire was reviewed by a panel of reproductive medicine specialists. Feedback from these clinicians was used to refine question wording, improve clarity, and ensure that items were relevant, appropriately framed, and suitable for the target population. Iterative amendments were made following this review process prior to finalisation of the survey.

Between 13 February 2025 and 29 September 2025, the survey was promoted on *X* and *Instagram* by trained healthcare professionals, who collectively have over 250,000 followers. A hyperlink was shared with participants directing them to the landing page of the online survey, which provided a participant information leaflet with a summary of the study. Participation was exclusively voluntary and participants were encouraged to read all information carefully and to ask the research team questions on anything that remained unclear. Following this page, participants were directed to a consent form. Participants were free to withdraw at any point during the questionnaire, up until the point of submission. There was no offer of incentive to complete the survey.

There were no pre-recruitment evaluations required to take part in this study. Participants were eligible for inclusion if they were males aged 18 and above, UK residents, and able to understand English and provide consent. The survey included 54 close ended questions, took 10–12 minutes to complete, and was designed for quantitative data interpretation (S1 File).

The survey consisted of four categories. First, respondents answered questions relating to their socio-demographic characteristics including age, ethnicity, parental status, the number of children they have, relationship status, sexual orientation, education level, and employment status. Secondly, participants were assessed on their knowledge of fertility. Following this, participants were asked questions evaluating their current reproductive aspirations, including their desired age to have their first and last child, as well as whether they would consider freezing their sperm to prevent ARFD. Finally, participants completed questions assessing their attitudes towards fertility, indicating the extent to which they agreed with statements reflecting personal and socioeconomic influences using a Likert scale.

### Statistical analysis

R version 4.5 was used for all statistical analysis. Shapiro-Wilk test was used to assess for normality and descriptive statistics subsequently presented as median and interquartile range (IQR). Circular proportion plots illustrating the distribution of responses were labelled with percentages for categories representing ≥3% of responses only, in order to preserve clarity.

### Ethical approval

This study was reviewed and approved by the Imperial College Research Ethics Committee (7000608) on 15 May 2024. All participants provided written informed consent after reviewing the accompanying patient information leaflet.

## Results

A total of 408 young men participated in the survey, of which 214 (52.5%) completed all components. Participants had a median age of 34 years (IQR 31–38) and were predominantly of white ethnicity, 86.8% (N = 249) (Table 1). Of those who responded, most young men reported they were either married (61.5%; N = 176), or cohabiting with a partner (21.0%; N = 60). Heterosexual men accounted for the majority of the cohort (95.5%; N = 275), with just 3.5% (N = 10) identifying as homosexual or bisexual. The majority of participants had received higher education, with 37.8% (N = 109) of participants reporting an undergraduate degree, 33.7% (N = 97) reporting a postgraduate degree, and 6.9% (N = 20) reporting a doctorate. A minority of responses were from young men who had GCSE or A Level qualifications as their highest level of education, accounting for a combined 8.3% (N = 24) of participants (Table 1). The majority of young men who responded reported being employed in full time work (85.4%; N = 246).

**Table 1:** Patient characteristics.

	N	Proportion (%)
**Age (Years)**		
18–25	6	2.3
26–30	53	20.4
31–35	109	41.9
36–40	65	25.0
41–45	21	8.1
46–50	6	2.3
**Ethnicity**		
Asian	12	4.2
Black	12	4.2
Indian	4	1.4
Mixed	4	1.4
Other	3	1.0
White	249	86.8
Would rather not say	3	1.0
**Relationship Status**		
Cohabiting with a partner	60	21
In a relationship	25	8.7
Married	176	61.5
Separated	2	0.7
Single	22	7.7
Widowed	1	0.3
**Sexual Orientation**		
Bisexual	2	0.7
Heterosexual	275	95.5
Homosexual	8	2.8
Would rather not say	3	1.0
**Education Level**		
GCSEs	11	3.8
A Levels	13	4.5
Diploma	11	3.8
Undergraduate degree	109	37.8
Postgraduate degree	97	33.7
Professional Qualification	23	8.0
Doctorate	20	6.9
No formal qualifications	4	1.4
**Employment Status**		
Employed (full time)	246	85.4
Employed (part time)	6	2.1
Self employed	26	9.0
Student	4	1.4
Unemployed	4	1.4
Homemaker	1	0.3
Retired	1	0.3

N numbers and corresponding proportions reflect only participants who provided a response to each question; denominators vary accordingly.

Young men without children accounted for 55.6% of respondents (N = 160), while 44.4% (N = 128) had children (Table 2). Participants with children had a maximum of four children (4.8%; N = 6), with the majority of respondents reporting only one child (52.8%; N = 66). Overall, most respondents (67.8%; N = 194) indicated that they would like to have children in the future (Table 2). Among existing parents, 48.0% (N = 61) wished to have further children, 18.9% (N = 24) were unsure, and 33.1% (N = 42) did not want additional children. Among childless participants, the majority expressed a desire to have children in the future (83.6%; N = 133), with 10.1% (N = 16) unsure, and 6.3% (N = 10) indicating no desire to have children in the future. Childless participants most frequently indicated a preferred age of 30–35 years for their first child (64.5%; N = 71), while 21.8% (N = 24) reported 36–45 years. Across all participants, 64.3% (N = 119) selected 36–45 years-old as the age they would like to have their last child, and 26.5% (N = 49) selected 30–35 years-old (Table 2). Almost half of participants indicated that they would consider freezing their sperm to prevent ARFD (44.8%; N = 95) (Table 2).

**Table 2:** Reproductive aspirations.

	N	Proportion (%)
**Do you have children?**		
No	160	55.6
Yes	128	44.4
**If yes, how many children do you have?**		
1	66	52.8
2	44	35.2
3	9	7.2
4	6	4.8
**Would you like to have children in the future?**		
No	52	18.2
Unsure	40	14.0
Yes	194	67.8
**Desired age to have first child (if childless)**		
20-24	1	0.9
25-29	10	9.1
30-35	71	64.5
36-45	24	21.8
46-55	1	0.9
Unsure	3	2.7
**Desired age to have last child**		
25-29	3	1.6
30-35	49	26.5
36-45	119	64.3
46-55	7	3.8
56-65	1	0.5
Unsure	6	3.2
**Would consider sperm freezing**		
No	69	32.5
Unsure	48	22.6
Yes	95	44.8

N numbers and corresponding proportions reflect only participants who provided a response to each question; denominators vary accordingly.

Table 3 outlines the responses of participants to a variety of questions evaluating their knowledge of fertility. Of those who answered, the majority were aware that paternal age may influence pregnancy rates (58.6%; N = 133) (Table 3) [13,15]. Similarly, the vast majority (85.5%; N = 194) understood that the chances of conception are influenced by body mass index (BMI) [29,30]. Participants most commonly identified male factor infertility as a leading cause of infertility in heterosexual couples (33.5%; N = 76), followed closely by ovulatory factors (31.3%; N = 71) and unexplained infertility (28.6%; N = 65), with fewer respondents selecting uterine (4.0%; N = 9) and tubal causes (2.6%; N = 6) (Table 3). Moreover, only a small proportion of the cohort (16.9%; N = 38) correctly identified that approximately 31–40% of infertility cases are primarily attributable to male factors, with responses generally overestimating this proportion (Table 3) [3,4]. Similarly, few participants correctly identified that heterosexual couples have between an 80–100% chance of conceiving after one year of unprotected sexual intercourse (14.2%; N = 32) [4].

**Table 3:** Participants’ knowledge of fertility.

Question	N	Proportion (%)
**Is the age of a man important for a couple’s chances to become pregnant?**		
Yes	133	58.6
No	76	33.5
Unsure	18	7.9
**Does the body mass index (BMI) of a man affect their ability to conceive?**		
Yes	194	85.5
No	14	6.2
Unsure	19	8.4
**What is the most common cause of infertility experienced by heterosexual couples?**		
Female problems with ovulation	71	31.3
Female problems due to damage of the fallopian tube	6	2.6
Female problems due to damage to the womb	9	4.0
Problems with the male	76	33.5
Unexplained	65	28.6
**What are the chances that a heterosexual couple, where the woman is 30 years of age, will become pregnant after one year of unprotected sexual intercourse?**		
<10%	1	0.4
10-19%	12	5.3
20-39%	40	17.7
40-59%	46	20.4
60-79%	80	35.4
80-100%	32	14.2
Unsure	15	6.6
**What percentage of infertility experienced by couples is primarily because of the male?**		
0-10%	5	2.2
11-20%	17	7.6
21-30%	46	20.4
31-40%	38	16.9
41-50%	52	23.1
51-60%	42	18.7
61-70%	17	7.6
71-80%	8	3.6
81-90%	0	0.0
91-100%	0	0.0
**At what age do you think men’s sperm quality/quantity begins to decline?**		
It does not decrease with age	22	9.7
30-35	45	19.9
36-40	40	17.7
41-45	55	24.3
46-50	19	8.4
51-55	27	11.9
56-60	3	1.3
61-65	4	1.8
66-70	0	0.0
>70	11	4.9
**What is a normal sperm count?**		
> 19 million	23	10.2
> 29 million	47	20.9
>39 million	94	41.8
>49 million	34	15.1
>59 million	27	12
**How often do men produce sperm?**		
Every 7–10 days	154	68.1
Every 11–20 days	13	5.8
Every 21–30 days	15	6.6
Every 31–40 days	7	3.1
Every 41–50 days	0	0.0
Every 51–60 days	3	1.3
Every 61–75 days	9	4.0
Every 3 months	25	11.1
Every 6 months	0	0.0
Every year	0	0.0
**If a man’s sperm count is reduced because of certain lifestyle factors and not because of a medical condition, do you think it is possible for the sperm count to be increased again?**		
Yes	200	88.9
No	4	1.8
Unsure	21	9.3
**Do you think that having children at a later age will impact the overall health of the child in the future?**		
Yes	108	48.0
No	78	34.7
Unsure	39	17.3
**If yes to the above question, from what age of the man do you think that having children will start to impact the overall health of the child in the future?**		
> 35 years old	26	24.1
> 45 years old	53	49.1
> 55 years old	21	19.4
> 65 years old	8	7.4

N numbers and corresponding proportions reflect only participants who provided a response to each question; denominators vary accordingly.

When participants were asked at what age sperm quality begins to decline, responses varied significantly (Table 3). The most frequently selected age range was 41–45 years (24.3%; N = 55), followed by 30–35 years (19.9%; N = 45) and 36–40 years (17.7%; N = 40). A smaller proportion of participants indicated 51–55 years (11.9%; N = 27), while 9.7% (N = 22) believed sperm quality does not decline with age. Few respondents selected ages beyond 60 years, with minimal responses for 61–65 (1.8%; N = 4), 56–60 (1.3%; N = 3), and none for 66–70 years. When asked to identify a normal sperm count, the most commonly selected response aligned with the WHO lower reference limit of >39 million (41.8%, N = 94), with remaining responses generally underestimating a normal sperm count (Table 3) [31]. The majority of participants believed that sperm production occurs every 7–10 days (68.1%; N = 154), while only a small proportion selected the range of 61–75 days (4.0%; N = 9), which is most consistent with current estimates of spermatogenesis duration (Table 3) [32]. The vast majority of participants (88.9%; N = 200) were aware that sperm count could be increased if it was originally reduced due to lifestyle factors, and not secondary to a medical condition [33–35]. Almost half the participants (48%; N = 108) recognised that advancing paternal age could subsequently impact the overall health of the child in the future (Table 3) [13,36,37]. Among those who identified such an association, almost half selected an age threshold of >45 years (49.1%; N = 53), making this the most frequently selected response (Table 3) [13,36,37].

Table 4 summarises the extent to which participants believed a variety of factors influenced male fertility using a Likert scale. In general, participants believed that the majority of presented factors influenced male fertility, with high proportions either ‘agreeing’ or ‘strongly agreeing’ that smoking (99.1%; N = 211), recreational drug use (95.3%; N = 203), and exposure to radiation/chemicals impacted male fertility (98.1%; N = 209) (Table 4) [38,39]. Participants were unsure whether the use of minoxidil or finasteride for hair loss prevention impacted male fertility, with 51.2% (N = 109) and 48.1% (N = 102) choosing neither agree nor disagree, respectively (Table 4) [40,41]. Similarly, 51.8% (N = 102) of participants neither agreed nor disagreed that COVID-19 exposure impacted male fertility [42]. Moreover, responses were split on whether protein supplements impacted male fertility, with 55.3% (N = 68) either agreeing or strongly agreeing, and 44.7% (N = 55) either disagreeing or strongly disagreeing (Table 4) [43].

**Table 4:** Participant’s perceptions of factors influencing fertility.

Factor	Strongly Agree N (%)	Agree N (%)	Neither Agree nor Disagree N (%)	Disagree N (%)	Strongly Disagree N (%)
Smoking	162 (76.1%)	49 (23.0%)	2 (0.9%)	0 (0.0%)	0 (0.0%)
Recreational drugs (e.g., cannabis/weed)	137 (64.3%)	66 (31.0%)	8 (3.8%)	2 (0.9%)	0 (0.0%)
Drinking more than 4 cups of coffee a day	47 (22.1%)	79 (37.1%)	65 (30.5%)	22 (10.3%)	0 (0.0%)
Protein supplements	17 (13.8%)	51 (41.5%)	0 (0.0%)	48 (39.0%)	7 (5.7%)
Anabolic steroid use (e.g., testosterone or testosterone boosting supplements)	127 (59.7%)	74 (34.7%)	12 (5.6%)	0 (0.0%)	0 (0.0%)
Diet high in sugar	75 (35.2%)	103 (48.4%)	22 (10.3%)	13 (6.1%)	0 (0.0%)
Use of Minoxidil (hair loss prevention)	29 (13.5%)	57 (26.8%)	109 (51.2%)	18 (8.5%)	0 (0.0%)
Use of Finasteride (hair loss prevention)	36 (17.0%)	61 (28.8%)	102 (48.1%)	13 (6.1%)	0 (0.0%)
Alcohol use of > 10 units a week	112 (52.6%)	94 (44.1%)	6 (2.8%)	1 (0.5%)	0 (0.0%)
Exposure to radiation/chemicals	152 (71.3%)	57 (26.8%)	4 (1.9%)	0 (0.0%)	0 (0.0%)
Wearing tight fit underwear frequently	73 (34.3%)	87 (40.8%)	22 (10.3%)	22 (10.3%)	9 (4.3%)
Frequent hot tub use	71 (33.3%)	82 (38.5%)	31 (14.6%)	23 (10.8%)	6 (2.8%)
Sexually transmitted infections, e.g., gonorrhoea/chlamydia/syphilis	119 (55.9%)	72 (33.8%)	13 (6.1%)	9 (4.2%)	0 (0.0%)
Stress	98 (46.0%)	102 (47.9%)	8 (3.8%)	4 (1.9%)	1 (0.4%)
Lack of exercise	77 (36.2%)	108 (50.7%)	17 (8.0%)	10 (4.7%)	1 (0.4%)
Frequent bicycling	41 (19.2%)	72 (33.6%)	56 (26.2%)	36 (16.8%)	9 (4.2%)
Use of phones/laptops	16 (7.5%)	58 (27.2%)	69 (32.4%)	58 (27.2%)	12 (5.7%)
Exposure to COVID-19	3 (1.5%)	35 (17.8%)	102 (51.8%)	57 (28.9%)	0 (0.0%)
Having diabetes	46 (21.6%)	113 (53.1%)	39 (18.3%)	13 (6.1%)	2 (0.9%)

Participants were asked to rate whether they thought the following factors had an impact on male fertility. N numbers and corresponding proportions reflect only participants who provided a response to each question; denominators vary accordingly.

**Fig 1:**
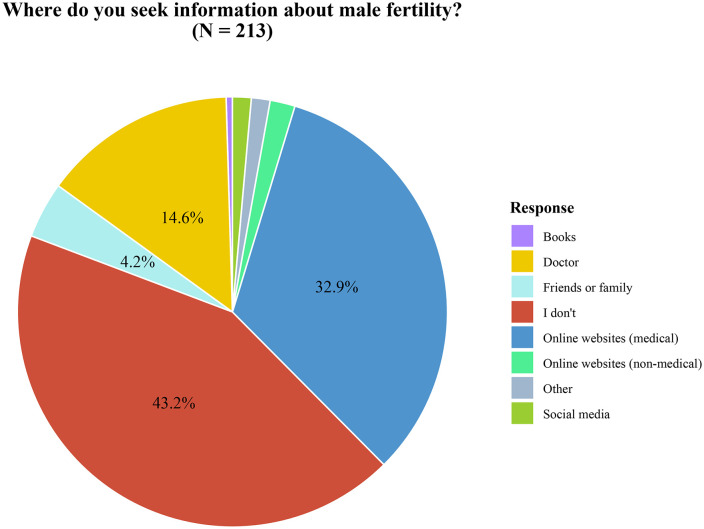
Circular proportion plot illustrating where participants seek information about male fertility. Books (purple); doctor (yellow); friends or family (light blue); does not seek information about male fertility (red); online websites (medical) (dark blue); online websites (non-medical) (light green); other (grey); social media (dark green).

Most young men (43.2%; N = 92) reported that they did not seek information about fertility (Fig 1). Amongst those who did seek information however, online medical websites were the most common source of information (32.9%; N = 70), followed by their doctor (14.6%; N = 31) (Fig 1). Seeking information from friends or family received a smaller proportion of responses (4.2%; N = 9), followed by non-medical websites (1.9%; N = 4). Social media, and other sources of information both received 1.4% of responses (N = 3). Finally, only one participant reported using books to gain information on male fertility, equating to 0.5% of responses (Fig 1).

**Fig 2:**
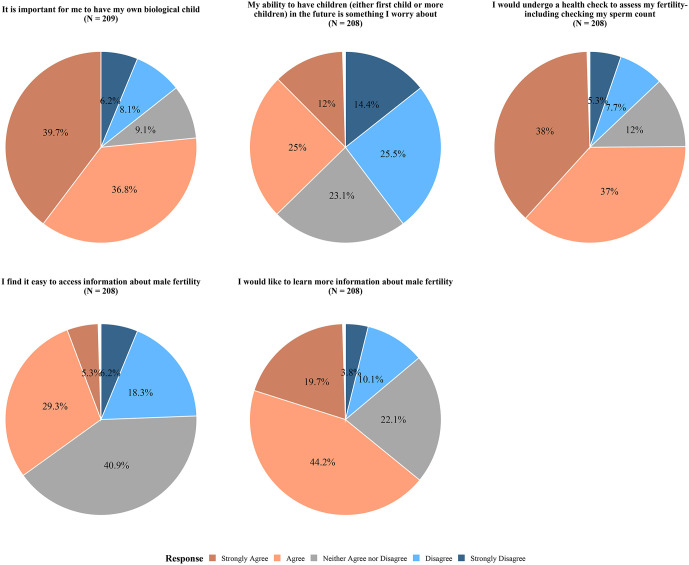
Circular proportion plots illustrating personal influences of attitudes towards fertility. Strongly agree (dark brown); agree (light brown); neither agree nor disagree (grey); disagree (light blue); strongly disagree (Dark blue).

Figure 2 summarises the personal influences which affect participants attitudes towards fertility, using a Likert scale. The majority of young men agreed (36.8%; N = 77) or strongly agreed (39.7%; N = 83), that it was important for them to have their own biological child (Fig 2). Responses were mixed when participants were asked whether their ability to have future children was something they worry about, with 25% (N = 52) choosing agree, 23.1% (N = 48) choosing neither agree nor disagree, and 25.5% (N = 53) choosing disagree (25.5%; N = 53) (Fig 2). When participants were asked if they would be willing to undergo a health check to assess their fertility, including checking their sperm count, the vast majority of respondents either agreed (37%; N = 77) or strongly agreed (38%; N = 79). Most participants neither agreed nor disagreed (40.9%; N = 85) that information on male fertility was easily accessible, while a smaller proportion agreed that they found such information easy to access (29.3%; N = 61) (Fig 2). The majority of participants indicated that they would like to learn more about male fertility, with agree and strongly agree receiving a combined 63.9% (N = 133) of responses, followed by neither agree nor disagree which received 22.1% (N = 46) of responses (Fig 2).

**Fig 3:**
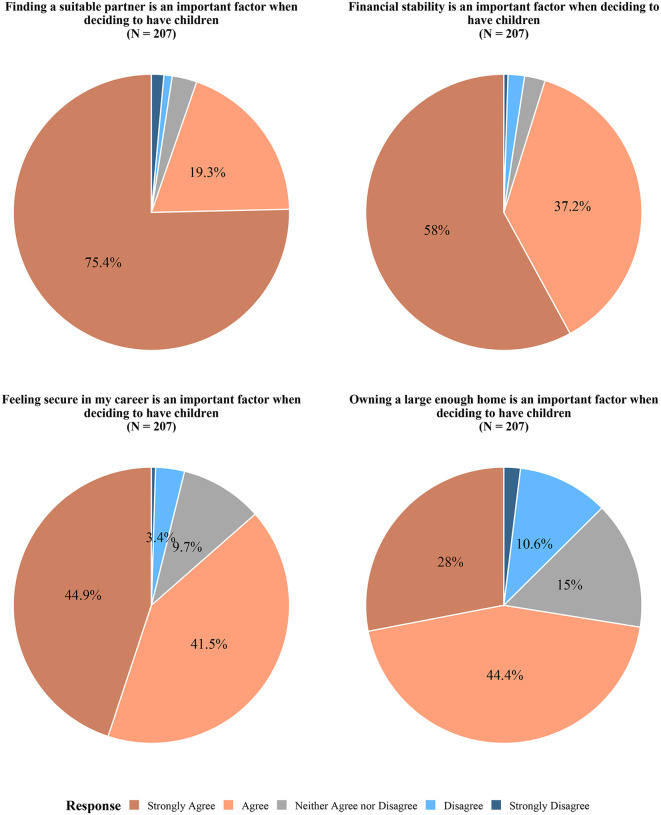
Circular proportion plots illustrating socioeconomic influences of attitudes towards fertility. Strongly agree (dark brown); agree (light brown); neither agree nor disagree (grey); disagree (light blue); strongly disagree (Dark blue).

With respect to socioeconomic influences affecting participants attitudes towards fertility, finding a suitable partner was considered an important factor when deciding to have children with 75.4% (N = 156) strongly agreeing and 19.3% (N = 40) agreeing with this statement. Similarly, participants either agreed or strongly agreed that financial stability (95.2%; N = 197) and feeling secure in their career (86.4%; N = 179) were important factors when having children. Finally, the majority (72.4%; N = 150) either agreed or strongly agreed that owning a large enough home is an important factor when deciding to have children (Fig 3).

## Discussion

This study represents the largest UK cohort of young men to report their knowledge, attitudes, and perceptions of male fertility to date. Our findings mirror trends observed in national demographic data, with young men expressing intentions to pursue fatherhood at increasingly older age, compared with previous decades [11,12,17]. We also demonstrate gaps in men’s understanding of male fertility, particularly regarding the contribution of male-factor infertility and key aspects of reproductive physiology, although there is a general desire amongst participants to gain more information regarding their own fertility. In addition, this survey identifies important personal and socioeconomic determinants of young men’s reproductive aspirations, offering insight into the underlying factors shaping contemporary family-planning decisions and providing an opportunity to inform more targeted public-health education of male fertility.

Compared with national representative census data from the Office for National Statistics (ONS), participants were broadly reflective of the wider UK population, although some important differences exist. The proportion of young white men was 86.8%, similar to the national estimate of 83.1% [44], and 95.5% of participants identified as heterosexual, aligning with ONS data reporting 93.0% of men as heterosexual [45]. However, participants had a higher education level compared to the general population, with 78.4% holding a degree level qualification or higher, compared to 33.8% nationally [46]. A higher proportion of young men were married (61.5% vs 49.5%) [47], and in full-time employment (85.4% vs 78%) [48]. The median age of participants was 34 years (IQR 31–38), closely matching the average paternal age at childbirth of 33.7 years, suggesting the sample captures young men at peak reproductive age [17]. Direct comparisons for parenthood status by age group are limited. While 66.8% of households are reported to have at least one child, this measure spans all age groups and is not directly comparable to our cohort, in which 44.4% reported having children [49]. This difference may reflect a younger population still in the process of family formation. Overall, while the cohort is comparable to the UK population across certain demographic domains, it appears skewed towards a younger, more highly educated, and socioeconomically advantaged group, which may limit the generalisability of the findings.

In general, young men reported a preference to have their first and last child between 30 and 45 years-old. Advancing paternal age has been associated with gradual changes in semen quality, including reductions in semen volume, progressive motility, and the proportion of morphologically normal sperm, as well as increased sperm DNA fragmentation [27,50]. Although the independent effect of paternal age on reproductive outcomes appears smaller than that of maternal age [16], evidence suggests that delayed fatherhood may contribute to reduced reproductive potential and adverse offspring outcomes [51,52]. This has been demonstrated in a donor-oocyte study, which reported that the odds of live birth decreased by 26% for every five year increase in paternal age beyond 25 years, suggesting that paternal ageing may have clinically meaningful consequences independent of maternal factors [53]. Given that our data suggests young men increasingly aspire to father children at older ages, these findings raise concerns that despite a strong preference for biological parenthood, an expanding gap between reproductive intentions and biological capability, may leave many couples struggling to achieve their intended family size or at increased risk of involuntary childlessness [11,12,26].

Importantly, the impact of delayed fatherhood must also be considered alongside the well-established impact of maternal age on reproductive outcomes, which can compound the reproductive challenges faced by couples. Increasing maternal age is associated with a progressive decline in ovarian reserve and oocyte quality, with an associated decline in pregnancy and live birth rates [54–56]. Indeed, predictive modelling has suggested that 14.3% of women who delay conception until 35 years-old would remain involuntarily childless, increasing to 34.8% among those delaying conception until 40 years-old [57]. Maternal age is arguably a stronger predictor of involuntary childlessness compared to paternal age, contributing to the increasing uptake of elective oocyte cryopreservation (EOC) as a strategy to mitigate the effects of ARFD [58,59]. However, advancing maternal age continues to negatively affect EOC outcomes, with age at cryopreservation a major determinant of oocyte yield and subsequent live birth success [60,61]. While women who undergo EOC at younger ages may improve their likelihood of achieving future pregnancy, most women currently elect to undergo this procedure at approximately 37 years-old, by which stage a degree of ARFD has already reduced their reproductive potential [62–64]. Consequently, while there is a pressing need to improve fertility awareness which has already been described [10], improving understanding of how advancing age affects the reproductive potential of both partners may help couples make more informed reproductive decisions, optimise their chances of achieving pregnancy, and reduce the risk of involuntary childlessness.

Notably, most young men indicated that they would be willing to consider sperm cryopreservation to mitigate the effects of ARFD. Although cryopreservation is associated with reductions in several semen parameters, clinical outcomes following the use of cryopreserved sperm appear broadly comparable to those achieved with fresh sperm in age-matched in-vitro fertilisation (IVF) cycles [65–67] However, evidence regarding the impact of cryopreserving semen at younger ages is limited, and it remains unclear whether earlier cryopreservation would provide meaningful benefits in mitigating ARFD. Consequently, while semen cryopreservation may offer a potential means to mitigate the impact of ARFD in the future, alongside wider patient education and counselling initiatives, further evidence is required to support the use of semen cryopreservation for this purpose.

The possibly over optimistic expectations of male fertility observed within this cohort may reflect the longstanding lack of fertility knowledge within the general population [26,68]. In a previous survey of women (N = 5,500), participants generally underestimated their reproductive potential, indicating that fertility decline begins earlier than documented in current literature [10]. By comparison, our data suggests young men may overestimate the extent of their reproductive potential, inaccurately believing that fertility decline begins at older ages than is supported by current evidence [69]. Semen quality has been shown to begin to decline in men ages 35 and over [69], with clinical manifestations, including a prolonged time to conception, becoming more pronounced in men 40 years and older [70,71]. More broadly, participants demonstrated gaps in their understanding of reproductive biology, suggesting that misconceptions extend beyond reproductive ageing alone.

It is perhaps surprising that overall knowledge of fertility was considered poor, despite the relatively high education attainment of participants compared to the general population, with 78.4% (N = 226) of our cohort receiving higher level education [72]. This may reflect the fact that most participants reported not actively seeking fertility information, and their mixed levels of concern about future childbearing may suggest that fertility is not currently a topic routinely discussed or prioritised. Reassuringly, young men expressed a strong interest in receiving additional fertility information, and many indicated they would agree to a health check which would assess their fertility, including checking their sperm count. Although universal semen analysis is unlikely to be feasible or cost-effective in presumed fertile men, routine health checks may nonetheless provide an important opportunity to deliver tailored fertility education and counselling [73]. Such an approach could help improve young men’s understanding of the timing and limits of their reproductive potential, support more informed reproductive decision‑making, and ultimately contribute to broader public health strategies aimed at integrating male fertility awareness into reproductive health policy. Moreover, as poor semen quality has been associated with broader health concerns, reproductive counselling may offer a holistic approach that benefits both fertility awareness and overall wellbeing [74].

Despite the increasing availability of fertility-related information through social media and other digital platforms, [75–77], only a small proportion of our participants reported using these platforms to access such information (1.4%, N = 3). This limited uptake may, in part, contribute to the lower participation observed in this male cohort (N = 408), compared with a similar survey advertised on social media by Kasaven *et al.* which had a significantly higher recruitment of female participants (N = 5,482) [10]. This perhaps suggests that young men engage differently with health information compared to women, particularly with respect to digital platforms and highlights the need for alternative, targeted strategies to improve male fertility awareness. In particular, opportunistic counselling during routine healthcare interactions, such as consultations in primary care, may represent a more effective approach to engaging young men in discussions around fertility and supporting informed reproductive decision-making.

Given the limited understanding of reproductive ageing amongst participants, it is important to consider the extent to which young men recognise the implications of advancing paternal age for offspring health [13]. Increasing paternal age has been associated with a range of adverse clinical outcomes in offspring, including the development of diabetes mellitus [78,79], congenital abnormalities [80,81], and haematological malignancies [82]. Moreover, children born to fathers aged 45 and over have a 65% higher risk of mortality within the first five years of life compared with those born to fathers aged 30–34 years-old, underscoring the clinical relevance of paternal age in shaping offspring outcomes [83]. However, in our present survey, respondents were divided in their views, with only 48% (N = 108) of respondents correctly identifying that increasing paternal age is associated with adverse child health outcomes. Among those that did recognise this association, only 49.1% (N = 53) correctly identified that the effects are evident from approximately 45 years of age [13,36,37]. Evidently, patient counselling should also focus on the long-term health effects of offspring born from ART, to support a strategy for more comprehensive family-planning decision making.

Participants demonstrated a generally good understanding of modifiable risks factors for reduced fertility and recognised that addressing lifestyle factors can improve semen quality. The majority of respondents (85.5%; N = 194) correctly identified that BMI affects the chances of conception. Moreover, participants consistently agreed that smoking, alcohol, and sexually transmitted infections all impact male fertility [38,84–86]. These findings contrast with those of Daulmer *et al.,* who found that survey respondents (N = 701) were able to identify only half (53.1%) of the modifiable risk factors for male fertility, despite similar baseline education levels in participants [26]. While this discrepancy with the findings of Daulmer *et al.* may indicate that public awareness of fertility has evolved over time, direct comparisons between studies should be made cautiously given differences in geographic setting, participant demographics, and sampling frameworks [26].

Participants were divided on whether minoxidil, finasteride, or protein supplements affect male fertility, with similar proportions agreeing and disagreeing. Minoxidil and finasteride are increasingly common treatments for hair loss, including androgenic alopecia, and are associated with significant improvements in hair retention with regular administration [87]. However, despite these benefits, a recent systematic review of murine models indicated that minoxidil and finasteride may impair epididymal function and subsequent semen quality, potentially impacting reproductive outcomes [41]. While evidence of impaired fertility in humans remains limited, a recent analysis of adverse drug event reports from the United States and Europe over the past 23 years, demonstrated that male infertility was associated with both finasteride and minoxidil usage, with reporting odds ratios of 73.90 (95%CI 66.70–81.87) (N = 469) and 3.30 (95%CI 2.04–5.31) (N = 17), respectively [88]. By contrast, the impact of protein supplementation, including creatine, on male fertility remains uncertain. In a cross-sectional study of 770 Danish men, protein supplementation was not associated with alterations in semen parameters [43]. However, mouse models suggest that creatine supplementation in creatine deficient individuals may improve semen quality and reproductive potential [89]. Consequently, although young men appear divided in their perceptions of protein supplementation, and high-quality evidence remains limited, current data suggest that protein supplementation does not appear to have an overtly detrimental impact on male reproductive potential.

In our present survey, nearly all participants (94.7%; N = 196) agreed that finding a suitable partner is an important factor when deciding to have children. This is consistent with similar surveys of women, in which the absence of a partner is consistently the most influential reason for consideration of elective oocyte cryopreservation [28], with one survey indicating that 63% of women would consider oocyte cryopreservation for this reason [10]. Notably, finding a suitable partner is not the only contributing factor when deciding to start a family amongst young men, as most respondents indicated that financial stability, job security, and owning a home of sufficient size are also important considerations. This is consistent with findings from a study of male and female university students in Hong Kong, where male respondents (N = 92) similarly emphasised the importance of career stability when deciding to start a family [20]. Furthermore, broader economic analyses by the organisation for economic co-operation and development (OECD) have identified weakened labour markets, characterised by poor job stability, long working hours, and limited career stability, as significant drivers of declining fertility across several countries [90]. Likewise, a Chinese study of 287 cities demonstrated an inverse association between house prices and birth rates [91]. Consequently, these data suggest that while public health initiatives may enhance young men’s understanding of their reproductive health, their family-building aspirations may be shaped by a wider set of socioeconomic conditions, that extend beyond individual-level fertility awareness.

While this study provides an updated and more comprehensive assessment of male knowledge, attitudes, and reproductive aspirations compared with earlier studies, there are several limitations which could be addressed in future studies [20,25,26,92]. Firstly, participants were recruited exclusively from the UK, and their views may not reflect those of young men in other countries. Secondly, although this represents the largest UK survey on male fertility to date, only 408 young men initially responded, and just 52.5% (N = 214) completed all components. Consequently, such an attrition may limit the generalisability of our findings to the wider UK male population. Furthermore, recruitment of participants using social media may have introduced selection bias, reflected by a higher proportion of participants having undertaken higher education compared to the national average [72]. As this survey explored perceptions and understanding of fertility, young men with a pre-existing interest in reproductive health may have been more inclined to participate, which should be considered when interpreting the wider applicability of these findings. Future studies should seek to include participants from a wider array of socioeconomic and ethnic backgrounds by adopting broader recruitment methods beyond social media participation. This would ensure findings reflect a more diverse viewpoint which is essential for planning future education initiatives. Additionally, although this survey was reviewed by reproductive medicine specialists, it was not subjected to formal psychometric evaluation or extensive pilot testing beforehand, thus limiting the reliability and construct validity of the survey as an assessment tool. Moreover, as all questions were close ended, further exploration of participants’ ideas and attitudes towards male fertility were limited. Qualitative methods, such as structured interviews, may better elucidate the underlying reasons for young men’s knowledge gaps and perceptions of fertility, although these approaches are inherently more time-intensive. Furthermore, although associations were observed between reproductive attitudes and societal trends, the cross-sectional nature of the survey prevents any causal interpretation, limiting the extent to which these findings can be generalised. It is also important to recognise the association between previous exposure of fertility services and subsequent understanding of male fertility. Thus, future studies should aim to include a subgroup analysis differentiating participants with previous exposure, so that stratification of knowledge across different cohorts of young men can be interpreted more accurately [26].

## Conclusion

Our findings reflect national trends indicating that young men increasingly intend to have children at older ages [11,12,17]. However, despite this shift, participants generally underestimated the impact of advancing paternal age on fertility outcomes. Given the relatively low utilisation of social media as a source of fertility information among young men, targeted strategies to support reproductive planning are required, such as opportunistic counselling during routine healthcare interactions. Generalisability of findings should be considered with caution, given that data may not be representative of diverse populations.

## Supporting information

S1 File Survey assessing male fertility awareness in the UK. Participants completed the questionnaire after reading a summary of the study and providing informed consent. The anonymous online survey was hosted on Qualtrics®.
(DOCX)
