# Supplementary material for: Exploring the knowledge, attitudes, and perceptions of young men towards fertility in the UK: A cross-sectional survey
Source: PLoS One. 2026 Jul 10;21(7):e0353073. doi: 10.1371/journal.pone.0353073 (PMC13353957; doi:10.1371/journal.pone.0353073)
Supplement: S1 File — Participants completed the questionnaire after reading a summary of the study and providing informed consent. The anonymous online survey was hosted on Qualtrics®. (DOCX) [file pone.0353073.s001.docx]

**Survey assessing male fertility awareness in the UK**

**There are three sections**

Section 1) About you

Section 2) Knowledge of fertility

Section 3) Perception of fertility

Section 4) Attitudes towards fertility

In total, the questionnaire will take you approximately 10-12 minutes to complete. You can pause and return to the questionnaire multiple times. You may skip questions or entire sections if you do not wish to provide a response.

**Section 1: About you**

**Age**

(Please state)

**Ethnicity**

*White/Asian/Black/Mixed/Would rather not say/ Other*

**Do you have children?**

*Yes (If yes, number)/No*

**Would you like to have children in the future?**

*Yes/No*

**What is your relationship status?**

*Single/In a relationship/Cohabiting with partner/Married/Separated/Divorced/Widowed*

**What is your sexual orientation?**

*Heterosexual/Bisexual/Homosexual/Would rather not say/Other*

**What is the highest degree/level of school education?**

*No formal qualifications/GCSEs/A levels/Diploma/Professional Qualification/Undergraduate degree/Postgraduate degree/Doctorate*

**What is your employment status?**

*Employed (full time)/Employed (part time)/Self-employed/Homemaker/Student/Retired/Unemployed*

**Section 2: Knowledge of fertility**

**Is the age of a man important for a couple’s chances to become pregnant?**

*Yes/No/Unsure*

**Does the body mass index (BMI) of a man affect their ability to conceive?**

*Yes/No/Unsure*

**What is the most common cause of infertility experienced by heterosexual couples?**

*Female problems with ovulation, Female problems due to damage of the fallopian tubes/ Female problems due to damage to the womb/problems with the male/ unexplained*

**What are the chances for a heterosexual couple, where the woman is 30 years of age, will become pregnant after one year of unprotected sexual intercourse?**

*<10%/10-19%/20-39%/40-59%/60-79%/80-100%/unsure*

**What percentage of infertility experienced by couples is primarily because of the male?**

*0-10%/11-20%/21-30%/31-40%/41-50%/51-60%/61-70%/71-80%/81-90%/91-100%*

**At what age do you think men’s sperm quality/quantity begins to decline?**

*It does not decrease with age/30-35/36-40/41-45/46-50/51-55/56-60/61-65/66-70/>70*

**What is a normal sperm count?**

*>19 million />29 million / >39 million / > 49 million/ >59 million*

**How often do men produce sperm?**

*7-10 days/11-20 days/21-30 days/31-40 days/41-50 days/51-60 days/61-75 days/every 3 months/every 6 months/every year*

**If a man’s sperm count is reduced because of certain lifestyle factors and not because of a medical condition, do you think it is possible for the sperm count to be increased again?**

*Yes/No/Unsure*

**Do you think that having children at a later age will impact the overall health of the child in the future?**

*Yes/No/Unsure*

**If yes to the above question, from what age of the man do you think that having children will start to impact the overall health of the child in the future?**

*>35/>45/>>55/>65*

**Please rate whether you think the following has an impact on male fertility:**

|  | Strongly disagree | Disagree | Neither agree, nor disagree | Agree | Strongly agree |
| --- | --- | --- | --- | --- | --- |
| **Smoking** |  |  |  |  |  |
| **Recreational drugs (e.g. cannabis/weed)** |  |  |  |  |  |
| **Drinking more than 4 cups of coffee a day** |  |  |  |  |  |
| **Protein supplements** |  |  |  |  |  |
| **Anabolic steroid use (e.g. testosterone or testosterone boosting supplements)** |  |  |  |  |  |
| **Diet high in sugar** |  |  |  |  |  |
| **Use of Minoxidil (hair loss prevention)** |  |  |  |  |  |
| **Use of Finasteride (hair loss prevention)** |  |  |  |  |  |
| **Alcohol use of > 10 units a week** |  |  |  |  |  |
| **Exposure to radiation /chemicals** |  |  |  |  |  |
| **Wearing tight fit underwear frequently** |  |  |  |  |  |
| **Frequent hot tub use** |  |  |  |  |  |
| **Sexually transmitted infections e.g. gonorrhoea/chlamydia/syphilis** |  |  |  |  |  |
| **Stress** |  |  |  |  |  |
| **Lack of exercise** |  |  |  |  |  |
| **Frequent bicycling** |  |  |  |  |  |
| **Use of phones/laptops** |  |  |  |  |  |
| **Exposure to COVID-19** |  |  |  |  |  |
| **Having diabetes** |  |  |  |  |  |

**Where do you seek information about fertility?**

I don’t/Online websites- medical/ Online websites- non medical/ Social media/ Friends or family/ Doctor/Books/Apps/Other

**Section 3: Perception of fertility**

**What is the desired age to have your first child? (If not yet had children)**

*20-24/25-29/30-35/36-45/46-55/56-65/66-75/unsure/NA*

**What is the desired age to have your last child?**

*20-24/25-29/30-35/36-45/46-55/56-65/66-75/unsure/NA*

**I would consider freezing my sperm to prevent age related fertility decline?**

*Yes/No/Unsure*

**Section 4: Attitudes toward fertility**

|  | Strongly disagree | Disagree | Neither agree, nor disagree | Agree | Strongly agree |
| --- | --- | --- | --- | --- | --- |
| **It is important for me to have my own biological child** |  |  |  |  |  |
| **My ability to have children (either first child or more children) in the future is something I worry about** |  |  |  |  |  |
| **I would undergo a health check to assess my fertility- including checking my sperm count** |  |  |  |  |  |
| **I find it easy to access information about male fertility** |  |  |  |  |  |
| **I would like to learn more information about male fertility** |  |  |  |  |  |

**How important are the following factors when deciding to have children:**

|  | Strongly disagree | Disagree | Neither agree, nor disagree | Agree | Strongly agree |
| --- | --- | --- | --- | --- | --- |
| **Finding a suitable partner** |  |  |  |  |  |
| **Financial stability** |  |  |  |  |  |
| **Feeling secure in my career** |  |  |  |  |  |
| **Owning a large enough home** |  |  |  |  |  |

Please leave any comments that you have for the research team : (free text box)

Thank you for taking part.
